# Surface/Interface Effects by Alkali Postdeposition Treatments of (Ag,Cu)(In,Ga)Se_2_ Thin Film Solar Cells

**DOI:** 10.1021/acsaem.1c02990

**Published:** 2021-12-20

**Authors:** Natalia M. Martin, Tobias Törndahl, Erik Wallin, Konstantin A. Simonov, Håkan Rensmo, Charlotte Platzer-Björkman

**Affiliations:** † Solar Cell Technology, Department of Materials Science and Engineering, Uppsala University, Uppsala, 751 21, Sweden; ‡ Solibro Research AB, Vallvägen 5, Uppsala, 756 51, Sweden; § Molecular and Condensed Matter, Department of Physics and Astronomy, Uppsala University, Uppsala, 751 21, Sweden

**Keywords:** thin film solar cells, ACIGS, alkali-PDT, composition analysis, HAXPES

## Abstract

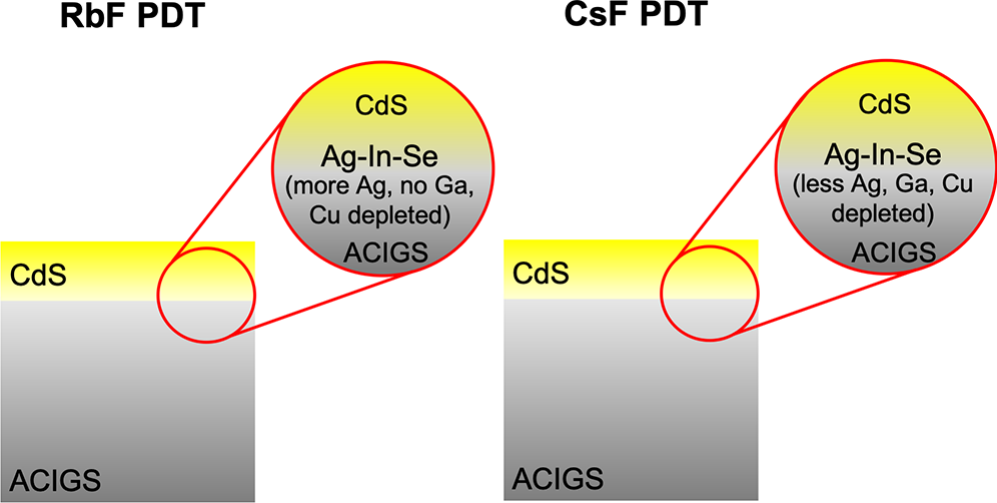

Ag alloying and the introduction of alkali elements through a postdeposition treatment are two approaches to improve the performance of Cu(In,Ga)Se_2_ (CIGS) thin film solar cells. In particular, a postdeposition treatment of an alkali metal fluoride of the absorber has shown a beneficial effect on the solar cells performance due to an increase in the open circuit voltage (*V*_OC_) for both (Ag,Cu)(In,Ga)Se_2_ (ACIGS) and CIGS based solar cells. Several reasons have been suggested for the improved *V*_OC_ in CIGS solar cells including absorber surface and interface effects. Less works investigated how the applied postdeposition treatment influences the ACIGS absorber surface and interface properties and the subsequent buffer layer growth. In this work we employed hard X-ray photoelectron spectroscopy to study the chemical and electronic properties at the real functional interface between a CdS buffer and ACIGS absorbers that have been exposed to different alkali metal fluoride treatments during preparation. All samples show an enhanced Ag content at the CdS/ACIGS interface as compared to ACIGS bulk-like composition, and it is also shown that this enhanced Ag content anticorrelates with Ga content. The results indicate that the absorber composition at the near-surface region changes depending on the applied alkali postdeposition treatment. The Cu and Ga decrease and the Ag increase are stronger for the RbF treatment as compared to the CsF treatment, which correlates with the observed device characteristics. This suggests that a selective alkali postdeposition treatment could change the ACIGS absorber surface composition, which can influence the solar cell behavior.

## Introduction

Solar cells based on Cu(In,Ga)Se_2_ (CIGS) thin film absorbers have shown high and stable efficiency values for both laboratory cells and industrial modules with a recent record cell efficiency of 23.4%.^1^ To reach closer to the theoretical maximum efficiency of around 33%, further reductions of optical and electrical losses are needed. Some recent works show that silver (Ag) alloying in CIGS to form (Ag,Cu)(In,Ga)Se_2_ (ACIGS) leads to higher device efficiencies as compared to similar CIGS devices without Ag.^2^ More, a postdeposition treatment (PDT) based on the alkali metal fluorides (i.e., KF, RbF, or CsF)^3−11^ applied after absorber formation has been studied intensively recently and is known to improve the efficiency in CIGS solar cells, mainly by an increase in the open circuit voltage, *V*_OC_.^12^ The exact role of the silver and/or alkali elements is discussed, but it is clear that interface and grain boundary effects are important in ACIGS solar cells. It is likely that a redistribution of the absorber elements near the surface region may occur during the alkali PDT, and recent studies showed the formation of an Alk–In–Se layer in CIGS solar cells subjected to an alkali metal fluoride PDT,^13−19^ while other studies, involving a PDT treatment without Se evaporation (as is the case of this work), did not find such a compound (see ref (7) and references therein).

Several previous works have investigated the surface properties of CIGS-based absorbers that have been exposed to different alkali metal fluorides. Some of the works have shown that the surface chemistry of CIGS is modified after PDT with KF, RbF, or CsF, and different surface electronic structures have been observed for different alkali metal fluoride treatments or type of absorber, although the improvement of the solar cell efficiency was very similar. It has thus been concluded that bulk effects (reduced bulk recombination) play an important role in the observed improvement of *V*_OC_ for CIGS solar cells in addition to surface effects (see for example ref (7) and references therein). In addition, the deposition of the CdS buffer layer by chemical bath deposition may alter the absorber surface properties and, to our knowledge, no previous works investigated how the ACIGS absorber near surface properties may change upon CdS buffer layer growth. Thus, the aim of the present work is to study how the real functional interface between alkali metal fluoride ACIGS and CdS may change with the choice of the alkali metal.

For this purpose, hard X-ray photoelectron spectroscopy (HAXPES) was employed to study the interfaces between CdS buffers and ACIGS absorbers, which have been exposed to two different alkali metal fluoride PDT processes, prior to the buffer layer deposition. The two different alkali-PDT processes are compared, RbF and CsF, to investigate how the choice of the alkali metal fluoride treatment affects the absorber and buffer/absorber interface. Also, CdS buffer layers of different thicknesses (9 and 18 nm) have been prepared in order to investigate how the interface develops during deposition. In particular, the physical and electronic properties of CdS/ACIGS interfaces in terms of intermixing, energy band alignment, and composition for a set of device relevant samples have been studied. Current–voltage and quantum efficiency measurements were additionally conducted on samples with the same thicknesses and preparations conditions and used to correlate the observed electronic and chemical changes to device properties.

## Experimental Section

### Sample Preparation and Device Characterization

Four ACIGS samples with 4 atomic % Ag content and PDT of CsF or RbF and a CdS buffer layer of either 9 or 18 nm, respectively, have been studied as summarized in Table 1. ACIGS solar cell samples were prepared using a thin film stack consisting of SLG/Mo/NaF/ACIGS + PDT/CdS/IZO/AZO. Mo was deposited by direct current (DC) magnetron sputtering in a commercial large-size tool on cleaned soda-lime glass (SLG), and substrates were subsequently cut down to 125 × 125 mm^2^ size for further processing. A NaF precursor layer (∼10 nm) was then deposited by thermal evaporation before the samples were transferred to the ACIGS deposition chamber. ACIGS films were prepared by coevaporation in a tool where deposition rates of the individual elements were controlled using a quadrupole mass spectrometer and a feedback control system to source powers. The films were deposited using a variant of a three-stage process involving a transition from Cu-poor growth, via a Cu-rich second stage to a Cu-poor final composition and experience a Ga-grading from back to front. The maximum substrate temperature during the process was ∼530 °C. The resulting films had an average composition of approximately Ag/I = 0.19; I/III = 0.85; Ga/III = 0.35 and a total thickness of 2.2 μm as determined by X-ray fluorescence (XRF). After ACIGS evaporation, samples were transferred in vacuo to another chamber where an alkali postdeposition treatment (PDT) was carried out involving either RbF or CsF. The alkali fluorides were deposited using thermal evaporation, and the deposition rate and film thickness was controlled using a quartz crystal microbalance (QCM). Samples were kept at a temperature of around 350 °C for the RbF PDT and 200 °C for the CsF PDT. No Se was coevaporated during the PDT process. Following the PDT process, the samples were flushed with water to remove any fluorine salts that were left on the surface of the absorber. Subsequently, a CdS buffer layer was deposited in a commercial large-size Stangl tool using a precursor solution containing cadmium sulfate, thiourea, and ammonia to a final thickness of either ∼9 or ∼18 nm as determined by the XRF calibrated against the profilometer. For the HAXPES measurements, after the buffer layer deposition, a part of each sample was packed and shipped to the synchrotron facility for analysis. For device samples, intrinsic ZnO (IZO) and 1% Al-doped ZnO (AZO) were deposited on the remaining parts of the samples by radio frequency (RF) and pulsed DC sputtering, respectively. Finally, an Al contact metal grid was deposited by thermal evaporation through a shadow mask.

**Table 1:** Summary of the (Ag,Cu)(In,Ga)Se_2_ Samples Investigated in This Experiment Together with the Bulk Composition Determined by XRF

sample no.	description	Ag/(Ag + Cu)	Ga/(Ga + In)	(Ag + Cu)/(Ga + In)	Cu/(Ga + In)
1	ACIGS + CsF PDT + 9 nm CdS	0.192	0.342	0.833	0.673
2	ACIGS + CsF PDT + 18 nm CdS	0.192	0.344	0.827	0.668
3	ACIGS + RbF PDT + 9 nm CdS	0.193	0.349	0.846	0.682
4	ACIGS + RbF PDT + 18 nm CdS	0.190	0.350	0.843	0.683

Current voltage (*I*–*V*) measurements were performed using an automized setup with a LOT Oriel Xe source, and quantum efficiency measurements were carried out using an Oriel IQE-200 setup. Samples were light soaked before *I*–*V* measurements.

### Hard X-ray Photoelectron Spectroscopy

HAXPES measurements were conducted at the GALAXIES beamline at the SOLEIL synchrotron radiation facility.^20^ The beamline, equipped with a double crystal monochromator (DCM), allowed tuning the excitation energy between 2.3 and 12 keV. A VG Scienta EW4000 energy electron analyzer and excitation energies *h*ν = 3 keV and *h*ν = 9 keV, respectively, were employed to record the photoemission spectra. A pass energy of 200 eV was used for all measurements yielding an analyzer resolution of 150 meV. The binding energy was calibrated by measuring the 4f spectrum of a grounded Au foil and setting the Au 4f_7/2_ binding energy to 84.0 eV, if not otherwise mentioned in the text. For the relative composition analysis, the HAXPES spectra has been fitted with a Voigt profile and a linear background using the Igor Pro software and taking into account the respective values for inelastic mean free path^21^ and photoionization cross-section,^22,23^ including the asymmetry parameters of photoelectric angular distributions.^23,24^ The analyzer transmission function was not taken into account which may introduce some error in the calculation of absolute values, but the aim of this study is to compare relative amounts between the investigated samples. Still, the bulk-like ACIGS composition determined from the HAXPES measurements yielded composition ratios similar to the ones determined from XRF, thus suggesting that the choice of method employed in this work for the relative composition analysis can still provide reliable results. Before the photoemission measurements, the samples were dipped into deionized water and dried by nitrogen gas before introducing them into the vacuum system of the HAXPES setup. A decreased photon flux has been employed to avoid beam damage.

## Results and Discussion

### Chemical and electronic properties of the buffer/absorber interface by HAXPES

To investigate the influence of different alkali PDT (CsF or RbF) and subsequent buffer layer deposition on the chemical and electronic structure of CdS/ACIGS interface, HAXPES measurements were performed.

**Figure 1:**
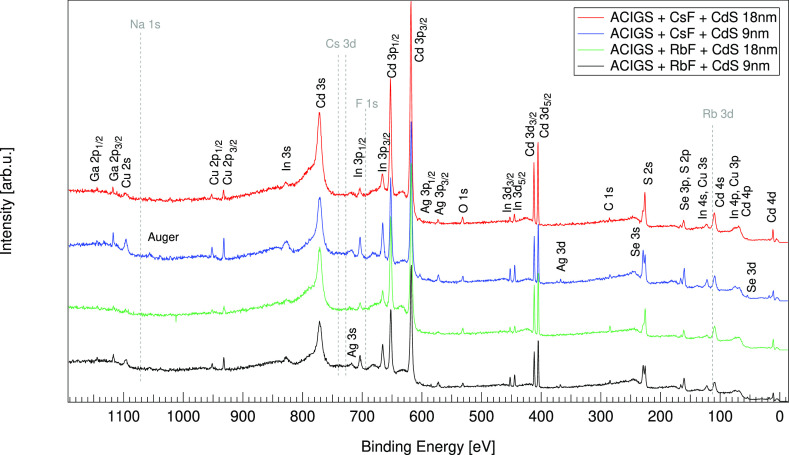
HAXPES survey spectra recorded at 9 keV for the PDT ACIGS/CdS sample series (PDT: CsF or RbF and CdS thicknesses: 9 or 18 nm, respectively). The most prominent lines are labeled and the spectra have been vertically offset for clarity. Dotted lines represent the expected positions for the Na 1s, Cs 3d, Rb 3d, or F 1s lines as indicated.

Figure 1 and Figure S1 (Supporting Information) illustrate the survey spectra of the investigated ACIGS samples using photon energies of 9 and 3 keV, respectively. The survey spectra measured at 3 keV, that is, with a lower bulk sensitivity, are very similar for the 18 nm thick CdS samples and show mainly the signals from the CdS buffer (Cd and S signals), while for the 9 nm thick CdS samples, some weak Cu, In, Se, and Ag signals are observed, in addition to the signals from CdS, suggesting that the interface between the ACIGS and CdS is probed at this photon energy for a thin (9 nm) CdS buffer (see also the high-resolution spectra, Figures S2 and S3). Only a weak Ga signal was observed on the 9 nm CdS samples, indicating Ga depletion from the near surface region and no alkali elements were detected. However, their presence at the ACIGS surface cannot be excluded as no reference samples without CdS were investigated and also as the alkali metal fluorides are likely to be present in low amounts at the interface. Additionally, some weak Zn signals are observed around 1020 eV for all samples, due to contamination during the CdS deposition process. Further, the presence of C and O contamination is observed, which is unavoidable on these types of samples during the fabrication process.

Some clear changes are observed in the survey spectra when the excitation energy has been increased to 9 keV, as compared to the measurements performed at 3 keV. As illustrated in Figure 1, Cu, In, Ga, Ag, and Se signals are observed for all samples, in addition to Cd and S signals, indicating that at 9 keV, being more bulk sensitive, both CdS and ACIGS near surface regions are clearly detected for all samples. A lower surface contamination of C and O is observed at 9 keV photon energy as also supported by the relative composition analysis (see Table S1), suggesting that the O and C contamination is contained within the CdS buffer layer. Similar to the measurements performed at 3 keV and discussed above, no signals from either Rb, Cs, or F are detected. The corresponding high-resolution spectra (see Supporting Information, Figure S4 and Figure S5) of individual core levels for the ACIGS samples with 9 nm CdS, in agreement with the survey spectra, show an increase of the absorber signals as expected due to a thinner CdS overlayer as compared to the samples with 18 nm CdS. Except for some intensity changes, no significant core level spectral differences are observed between the spectra recorded for the 9 nm vs 18 nm thick CdS samples.

In addition, the spectral shape and width of the main absorber and buffer signals do not change between the applied alkali metal fluoride treatments (CsF or RbF) or CdS buffer layer thickness (9 or 18 nm) for all investigated samples as Voight profiles with identical relative Gaussian and Lorentzian widths for a particular line were used to fit the spectra at each photon energy (see Figures S2, S3, S4, and S5, Supporting Information). This suggests that the chemical state around the Ag, Cu, In, Ga, and Se atoms is not significantly altered between the postdeposition treatment of either CsF or RbF and not influenced by the thickness of the CdS buffer layer. Further, no significant binding energy shifts have been observed between the investigated samples (see Table 2 and Table S2, Supporting Information), supporting the assumption of a similar chemical environment around the absorber elements for all investigated samples. However, the formation of an Alk–In–Se interlayer as previously reported,^13,14,16−19^ cannot be ruled out based on the current investigations, as reference samples without the alkali PDT have not been investigated in the present work. It is worth noting that such an interlayer formation has mainly been reported for PDT processes involving Se evaporation, while studies in which alkali metal fluoride deposition was performed in the absence of Se (as it is the case in this work) did not show significant chemical changes.

**Table 2:** Binding Energy Positions [eV] Recorded with a Photon Energy of 9 keV for the ACIGS Samples Investigated^a^

sample	Cu 2p_3/2_	In 3d_5/2_	Ga 2p_3/2_	Se 3s	Ag 3d_5/2_	Cd 3d_5/2_	S 2s
ACIGS + CsF PDT + 18 nm CdS	932.37	444.78	1117.80	229.42	367.84	405.20	225.99
ACIGS + CsF PDT + 9 nm CdS	932.34	444.80	1117.76	229.41	368.00	405.20	226.04
ACIGS + RbF PDT + 18 nm CdS	932.25	444.77	1117.79	229.32	367.97	405.20	225.98
ACIGS + RbF PDT + 9 nm CdS	932.31	444.78	1117.80	229.40	367.89	405.20	226.02

a The binding energy scale has been aligned to the Cd 3d_5/2_ peak position at 405.2 eV to exclude charging and surface/interface work function effects. An experimental uncertainty of 0.15 eV shall be considered for all reported binding energy values.

**Figure 2:**
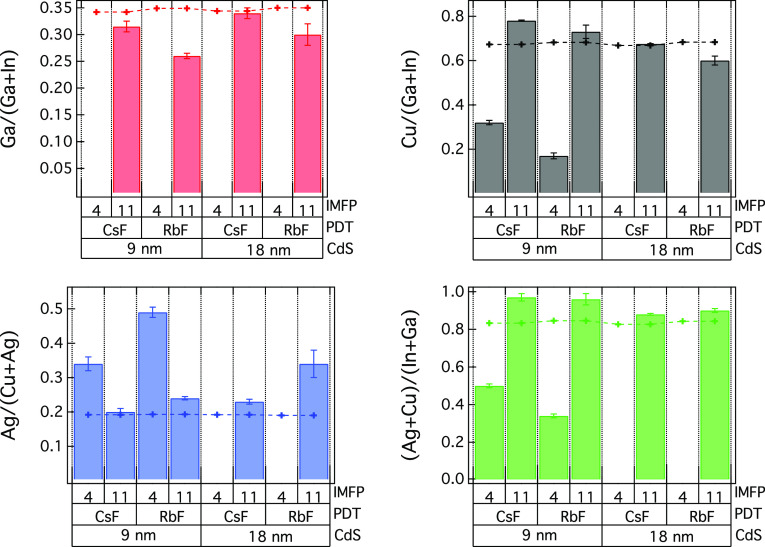
Relative composition analysis for the PDT ACIGS/CdS sample series investigated in this work as determined from HAXPES. The results are shown at both 3 keV (IMFP ∼ 4 nm) and 9 keV (IMFP ∼ 11 nm) and for both CsF and RbF treated samples. The results for the CdS buffer layer thickness of 9 and 18 nm are given. The XRF bulk composition is also included for comparison (+ symbols, dotted lines are used only to guide the eyes).

To compare the influence of the different alkali PDT processes and buffer layer thickness on the absorber composition at the buffer/absorber interface region, a relative composition analysis was performed as illustrated in Figure 2 and Table 3. The ratios are calculated based on the peak areas for the different components and divided by the IMFP and cross section (including asymmetry). Probing depths of ∼12 nm and ∼33 nm (∼3 × IMFP) were calculated from the average inelastic mean free path (IMFP) of absorber core levels at 3 and 9 keV, respectively. As observed in the survey spectra, and also discussed above, CdS is probed at the lowest probing depth (3 keV photon energy) for samples with 18 nm CdS, while at 9 keV the ACIGS near surface region is probed. The small ACIGS contributions observed at 3 keV for the 18 nm CdS samples (see Figure S2, Supporting Information) have been omitted in the composition analysis.

**Table 3:** Relative Composition Analysis for the PDT ACIGS/CdS Sample Series As Determined from HAXPES Measurements at Both 3 and 9 keV^a^

	Ga/(Ga + In)		Cu/(Ga + In)		Ag/(Cu + Ag)		(Ag + Cu)/(Ga + In)	
Sample	3 keV	9 keV	3 keV	9 keV	3 keV	9 keV	3 keV	9 keV
ACIGS + CsF PDT + 18 nm CdS		0.34 ± 0.01		0.675 ± 0.001		0.23 ± 0.007		0.88 ± 0.005
ACIGS + CsF PDT + 9 nm CdS		0.315 ± 0.01	0.32 ± 0.01	0.78 ± 0.003	0.34 ± 0.02	0.2 ± 0.01	0.50 ± 0.01	0.97 ± 0.02
ACIGS + RbF PDT + 18 nm CdS		0.30 ± 0.02		0.60 ± 0.02		0.34 ± 0.04		0.9 ± 0.01
ACIGS + RbF PDT + 9 nm CdS		0.260 ± 0.005	0.17 ± 0.013	0.73 ± 0.03	0.49 ± 0.015	0.24 ± 0.005	0.34 ± 0.01	0.96 ± 0.03

a The following core levels were used to calculate the ratios: Cu 2p_3/2_, Ga 2p_3/2_, In 3d_5/2_, Ag 3d_5/2_.

Compositional differences are observed for all investigated ratios (Ga/(Ga + In), Cu/(Ga + In), Ag/(Cu + Ag), (Ag + Cu)/(Ga + In), and (Se + In)/(Cu + Ga)) as a function of probing depth, indicating a nonconstant distribution of the elements near the ACIGS/CdS interface. An Ag–Ga anticorrelation is observed as previously reported.^25^ All samples are Cu, Ga poor and In, Se, Ag rich at the interface as compared to XRF measurements representing average stoichiometries throughout the bulk. Previously, a Cu and Ga depleted CIGS surface was reported when an alkali PDT was employed.^3,26,27^ The measurements also show that there is almost no Ga present at the surface for all samples, while still small amounts of Cu are present, even though in lower concentration than thorough the bulk material. It is likely that GaF_*x*_ and InF_*x*_ formation takes place after Alk-PDT as previously reported by Valdes et al.^28^ for KF PDT of CIGS. However, GaF_*x*_ species formed during the KF PDT of ACIGS have previously been shown to be dissolved by the ammonia solution during the subsequent chemical bath deposition of CdS,^29^ which may partly explain the suppression of Ga at the surface. Comparing the CsF and RbF treated samples, the RbF treated sample is more Ga and Cu poor than the CsF treated sample for both CdS buffer thicknesses, which may likely be due to the different PDT processing temperatures for RbF vs CsF, that is, 350 °C vs 200 °C, respectively. A higher Ga/(Ga + In) ratio was determined for the CsF treated samples near the ACIGS/CdS interface, while no significant difference was determined in the bulk composition of the samples as shown in Table 1 (independent of alkali-PDT or CdS thickness). Similarly, an increased Cu/(Ga + In) ratio is observed at the near CdS/ACIGS interface for the CsF treated sample as compared to the RbF sample, while an inverse trend is observed for the Ag/(Ag + Cu) ratio. Compositional changes at the near absorber/buffer interface are likely to influence the device behavior, and a comparison to electrical properties will be discussed more below. It is worth mentioning that the composition values determined for bulk-like ACIGS from the HAXPES are similar to the values determined by XRF and shown in Table 1, thus strengthening the fact that a reliable compositional analysis may be obtained thorough the method employed in this work.

Furthermore, the valence band (VB) spectra were recorded by HAXPES in order to obtain information about how the electronic properties (e.g., valence band alignment) at the ACIGS/CdS interface may change between the applied alkali-PDT. The VB spectra recorded for the investigated samples (see Figure S6, Supporting Information) are composed of a mixture of the VBs of CdS and ACIGS, and can be affected by various interface effects (e.g., Fermi-level pinning, defects, and lattice mismatch). To compare the onset of the VB structure of all samples directly excluding charging and surface/interface work function and doping effects, the position of the Cd 4d core level at ∼11 eV is used as the binding energy reference for all spectra as all samples have a CdS top layer. Similar results are observed when the position of In 3d was used as a reference (not shown).

The VB spectra recorded for the investigated samples show no significant differences between the CsF- or RbF-treated ACIGS samples suggesting that the valence band alignment is not significantly altered between the applied PDT of CsF or RbF. As shown in Figure S6, the spectra have been normalized to the Cd 4d peak to exclude any influence of the buffer layer thickness. The small shift for the 18 nm thick CdS as compared to the 9 nm thick CdS at 9 keV photon energy (Figure S6b, inset), independent of the alkali treatment might be related to the fact that at this photon energy we are probing different compositions within ACIGS which may impact the band gap and thus the valence band position. The VB spectra obtained by subtracting the 9 nm thick CdS from the 18 nm thick CdS at 9 keV are also shown in an attempt to subtract out the CdS buffer contribution to the VB spectra and obtain information on the underlying absorber VB contribution. Still no difference between the CsF or RbF treated samples was observed, thus suggesting a similar VB for the ACIGS absorbers exposed to either RbF or CsF. In addition, no binding energy shift of the absorber core levels is observed for the fixed binding energy position of the buffer core levels between the investigated samples (see Table 2 and Table S2, Supporting Information).

It is known that the surface composition may affect the band gap of ACIGS absorbers,^30^ and an increase in the band gap has been reported with increasing Ag content. It has also been reported that Ag alloying systematically lowers both the CBM and VBM levels of ACIGS.^30^ Thus, the observed Ag enrichment at the surface of the absorber for both CsF and RbF PDT, would influence the valence band contributing to the small shift in Figure S6b) (i.e., VBM shifts away from the FL as Ag content increases). Still the larger Ag enrichment for the RbF treated sample is not reflected as a shift in the VB spectra likely due to the small difference as compared to that for the CsF sample and also as the 9 keV measurements are more sensitive to the region below the surface.

Also, the formation of a novel compound (i.e., Ag–In–Se–Alk) at the ACIGS/CdS interface that could also influence the surface bandgap of ACIGS as it was previously reported for KF PDT of CIGS^31,32^ cannot be excluded. No shift in the effective band gap as determined from quantum efficiency measurements has been observed between the investigated samples (see Figure S7, Supporting Information) indicating that the suggested band gap widening is limited to the surface of the ACIGS absorber.

### Device Performance by *I*–*V* Measurements

**Figure 3:**
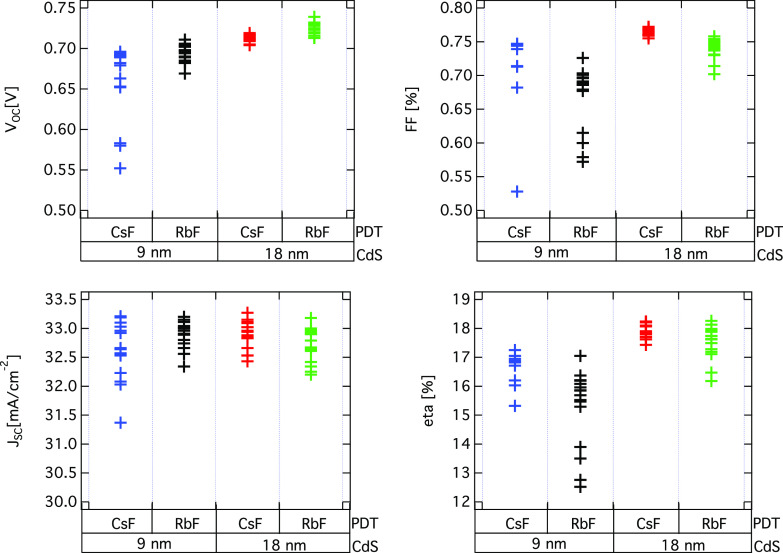
Photovoltaic properties for the solar cells fabricated from the PDT-ACIGS/CdS samples investigated in this work (PDT, CsF or RbF; and CdS thicknesses, 9 or 18 nm, respectively). The samples consist of the following cell structure: i:ZnO/ZnO:Al/CdS/PDT-ACIGS/Mo/SLG. (*V*_OC_, open-circuit voltage [V]; FF, fill factor [%]; *J*_sc_, short-circuit current density [mA/cm^2^]; eta, conversion efficiency [%]).

To investigate the impact of the ACIGS near the surface composition on the device performance, current–voltage (*I*–*V*) measurements were carried out on identical samples which have been processed into devices. The *I*–*V* curves for the devices prepared from the samples investigated in this work are presented in Figure S8 and the *I*–*V* characteristics are presented in Figure 3. The present results reveal some very good efficiencies for thinner CdS buffers as 18 nm thick CdS is close to the optimum for PDT CIGS in terms of efficiency. Whereas all investigated samples show very good performance, the results show a clearly better open circuit voltage (*V*_OC_) level for RbF as compared to that for CsF treated ACIGS samples. Also, the results show that the *V*_OC_ degrades with thin CdS for both PDT employed (CsF and RbF), whereas the fill factor (FF) follows an opposite trend. There is also a clear indication that the thinner CdS samples have better performance when a CsF PDT is employed as compared to RbF PDT. However, sputter induced changes of the CdS buffer, especially for the thin CdS (9 nm) layer, are possible during subsequent deposition of the window layer and thus may influence the observed device properties. Also, the short circuit current (*J*_SC_) seems to be very similar for 9 vs 18 nm samples, suggesting that there are no significant optical losses in the thin CdS samples, as also supported by the similar quantum efficiency results shown in Figure S9.

A comparison with the HAXPES results presented above indicates that there may be a correlation between the observed compositional changes and device properties for the CsF and RbF treated samples. The higher *V_*OC*_* for RbF as compared to CsF treated samples could possibly be connected to higher Ag surface content (thus higher acceptance of alkali elements in the absorber^33^ and lower VBM expected), together with lower Cu content (known to promote band gap widening in CIGS). Still, no significant difference in the VB spectra has been observed between the RbF and CsF treated samples as discussed above, which might be masked by the increased bulk sensitivity of the HAXPES measurements, and thus the shift cannot be resolved. Further, an increased Ag enrichment at the surface for the RbF treated sample may change the conduction band alignment at the ACIGS/CdS interface and reduce interface recombination as it has been reported for CIGSe devices with the addition of Ag.^25,30^ It is likely that a combination of the above may contribute to the observed differences in the device properties between the CsF- and RbF-treated samples.

Thus, the results indicate that the surface composition of ACIGS as modified by the applied PTD may impact the device characteristics. However, we cannot exclude that bulk effects (such as reduced bulk recombination) play a role in the observed improvement in *V*_OC_ and not just surface effects.

## Conclusions

Ag alloying and the introduction of alkali elements through a postdeposition treatment, PDT, are two approaches to increase the efficiency of CIGS solar cells. Using HAXPES, we have studied the CdS/ACIGS interface region of the ACIGS absorber material which was exposed to different alkali metal fluorides (RbF or CsF) PDT. The employed photon energies (3 and 9 keV) allowed probing at and below the 9 and 18 nm thick CdS buffers deposited on the PDT ACIGS absorbers.

The results show that the ACIGS near surface composition seems to change depending on the applied PDT process. In particular a Ag and In surface enhancement (decrease in Cu and Ga content) is observed for all samples. When the different PDT processes are compared, a decreased Cu and Ga content and slightly higher Ag content at the surface of the RbF treated samples, as compared to the CsF treated samples, is linked to increased *V*_OC_. The results give insights into how selective alk-PDT could change the ACIGS surface composition, which is likely to influence the solar cell behavior.

## Supporting Information Available

Supporting Information contains Figures S1–S9 and Tables S1–S2. The Supporting Information is available free of charge at https://pubs.acs.org/doi/10.1021/acsaem.1c02990.

Relative composition analysis for the (C+O)/ACIGS and (C+O)/CdS at both 3 and 9 keV; binding energy positions [eV] recorded with a photon energy of 3 keV for the ACIGS samples investigated; additional Figures S1–S9 (PDF)
