# Supplementary material for: Surface/Interface Effects by Alkali Postdeposition Treatments of (Ag,Cu)(In,Ga)Se2 Thin Film Solar Cells
Source: ACS Appl Energy Mater. 2021 Dec 20;5(1):461–8. doi: 10.1021/acsaem.1c02990 (PMC8790805; doi:10.1021/acsaem.1c02990)
Supplement: Supplementary file 1 — ae1c02990_si_001.pdf [file ae1c02990_si_001.pdf]

# Supporting Information

# Surface/interface effects by alkali post deposition treatments of (Ag,Cu)(In,Ga)$Se_2$ thin film solar cells


Natalia M. Martin,*,† Tobias Törndahl,† Erik Wallin,‡,¶ Konstantin A. Simonov,§,‖ Håkan Rensmo,§ and Charlotte Platzer-Björkman†

†*Solar Cell Technology, Department of Materials Science and Engineering, Uppsala University, Uppsala, 751 21, Sweden*

‡*Solibro Research AB, Vallvägen 5, Uppsala, 756 51, Sweden*

¶*Present address: EVOLAR AB, Uppsala, 756 51, Sweden*

§*Molecular and Condensed Matter, Department of Physics and Astronomy, Uppsala University, Uppsala, 751 21, Sweden*

‖*Present address: Swerim AB, Department of Materials and Process Development, Box 7047, Kista, 164 07, Sweden*

E-mail: Natalia.Martin@angstrom.uu.se


Figure S1 shows the HAXPES survey spectra of the investigated sample series recorded using 3 keV photon energy. At this energy, mostly CdS signals are observed as expected from the CdS overlayer. In addition, for the samples with 9 nm thick CdS, some absorber signals are observed indicating that the CdS layer thickness is below the probing depth of 12 nm ($\sim 3\times$IMFP). The dotted lines indicate positions of expected Na 1s, Cu 2p, Cs 3d,

Rb 3d or F 1s signals. No Rb or F signals are observed on any of the investigated samples. See main text for details.

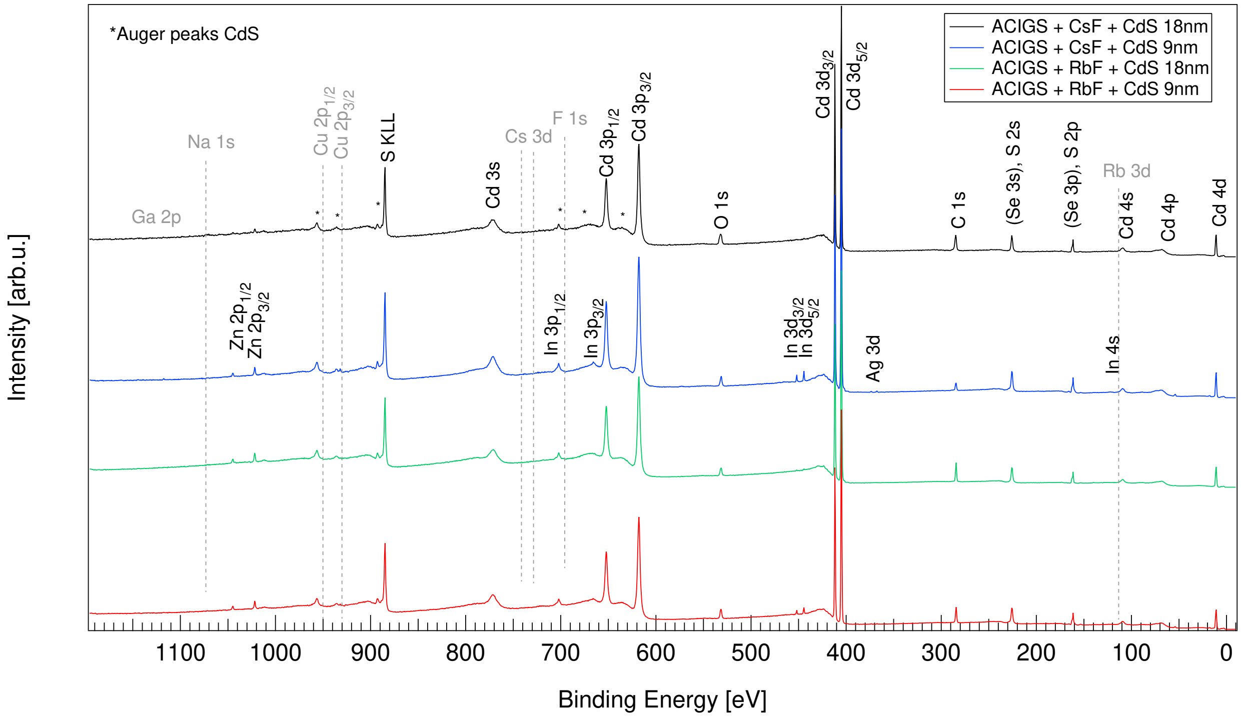


Figure S1: HAXPES survey spectra recorded at 3 keV for the PDT-ACIGS/CdS sample series (PDT: CsF or RbF and CdS thicknesses: 9 or 18 nm, respectively). The most prominent lines are labeled and the spectra have been vertically offset for clarity. Dotted lines represent the expected positions for the Na 1s, Cu 2p, Cs 3d, Rb 3d or F 1s lines as indicated.

Figures S2 and S3 show the high resolution photoemission spectra of the individual core-levels recorded at 3 keV photon energy for the investigated PDT-ACIGS/CdS samples. The weak Ga component seen towards the lower edge of the spectra has been missed during the measurements and assumed to be negligible as also indicated by the survey spectra above. To fit the spectra, a linear background and Voigt profiles of identical Gaussian and Lorentzian widths for a particular line were employed. The binding energy scale has been aligned to the Cd $3d_{5/2}$ peak position at 405.2 eV to exclude charging and work function effects. The broadening of the O 1s and C 1s core level spectra observed for S1 are due to additional chemical species around the O and C atoms which are out of the scope of this paper.

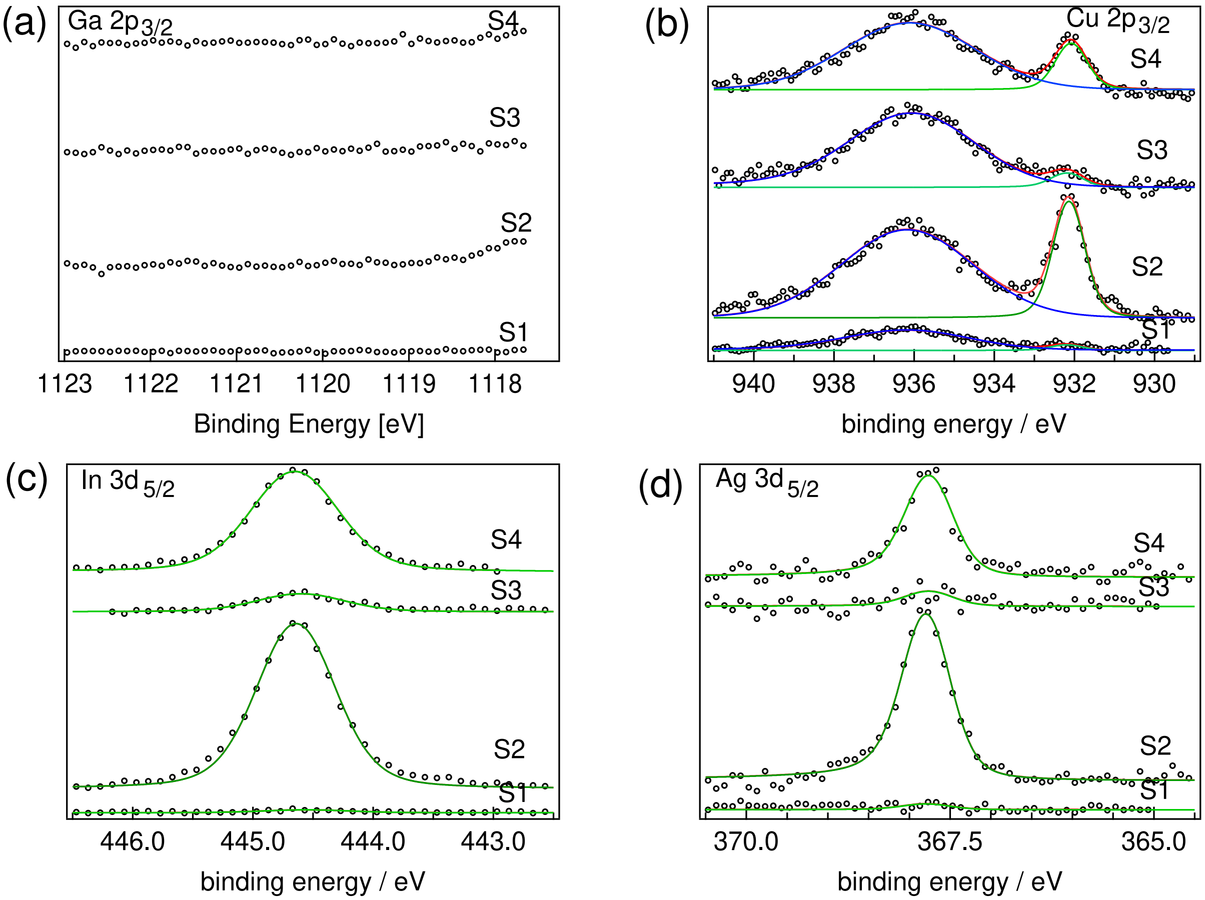


Figure S2: HAXPES data of CdS on PDT ACIGS samples, recorded with an excitation energy of 3 keV: a) Ga $2p_{3/2}$; b) Cu $2p_{3/2}$; c) In $3d_{5/2}$; d) Ag $3d_{5/2}$. The broad (blue) component observed in Cu 2p for all samples is an Auger component from CdS as it was previously been observed for CdS reference sample. The spectra are displayed with the respective fits with Voigt profiles and a linear background subtracted. S1: ACIGS + CsF PDT + 18 nm CdS; S2: ACIGS + CsF PDT + 9 nm CdS; S3: ACIGS + RbF PDT + 18 nm CdS; S4: ACIGS + RbF PDT + 9 nm CdS

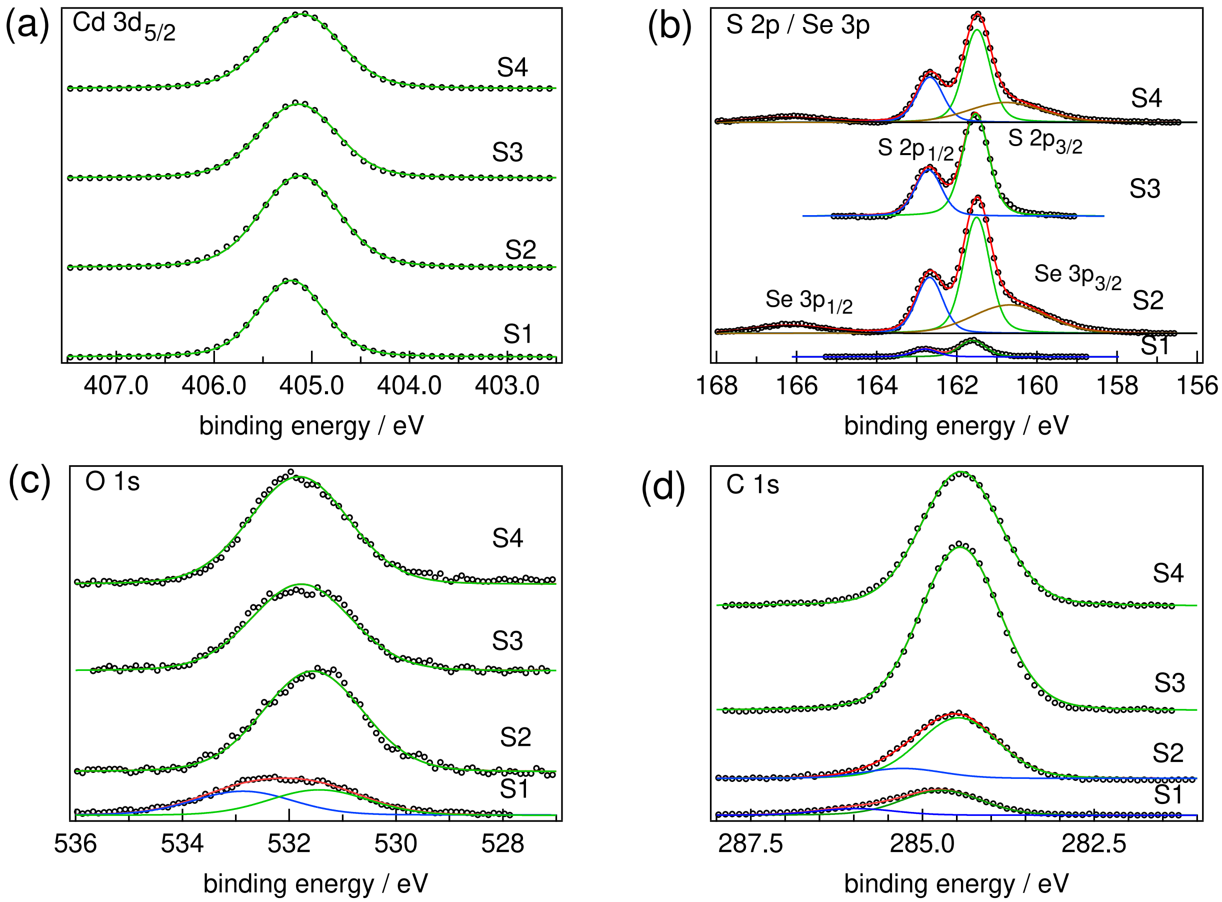


Figure S3: HAXPES data of CdS on PDT ACIGS samples, recorded with an excitation energy of 3 keV: a) Cd $3d_{5/2}$; b) Se 3p/ S 2p; c) O 1s; d) C 1s. The spectra are displayed with the respective fits with Voigt profiles and a linear background subtracted. S1: ACIGS + CsF PDT + 18 nm CdS; S2: ACIGS + CsF PDT + 9 nm CdS; S3: ACIGS + RbF PDT + 18 nm CdS; S4: ACIGS + RbF PDT + 9 nm CdS

Figures S4 and S5 show the high resolution photoemission spectra of the individual core-levels recorded at 9 keV photon energy for the investigated PDT-ACIGS/CdS samples. To fit the spectra, a linear background and Voigt profiles of identical Gaussian and Lorentzian widths for a particular line were employed. The binding energy scale has been aligned to the Cd $3d_{5/2}$ peak position at 405.2 eV to exclude charging and work function effects. Similar results are obtained when In $3d_{5/2}$ is used for binding energy calibration.

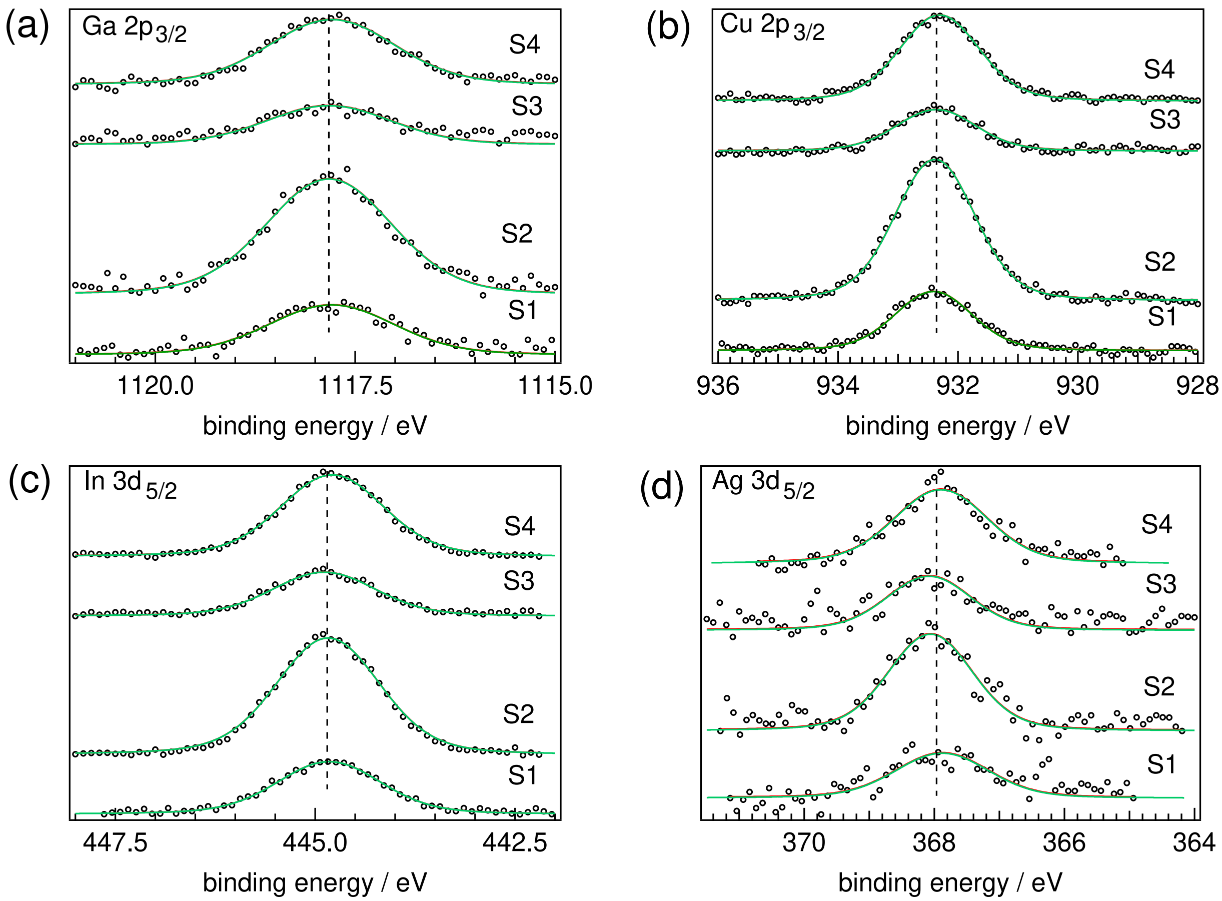


Figure S4: HAXPES data of CdS on PDT ACIGS samples, recorded with an excitation energy of 9 keV: a) Ga $2p_{3/2}$; b) Cu $2p_{3/2}$; c) In $3d_{5/2}$; d) Ag $3d_{5/2}$. The spectra are displayed with the respective fits with Voigt profiles and a linear background subtracted. S1: ACIGS + CsF PDT + 18 nm CdS; S2: ACIGS + CsF PDT + 9 nm CdS; S3: ACIGS + RbF PDT + 18 nm CdS; S4: ACIGS + RbF PDT + 9 nm CdS

Table S1 contains the relative composition analysis for the (C+O)/ACIGS and (C+O)/CdS at both 3 and 9 keV, respectively.

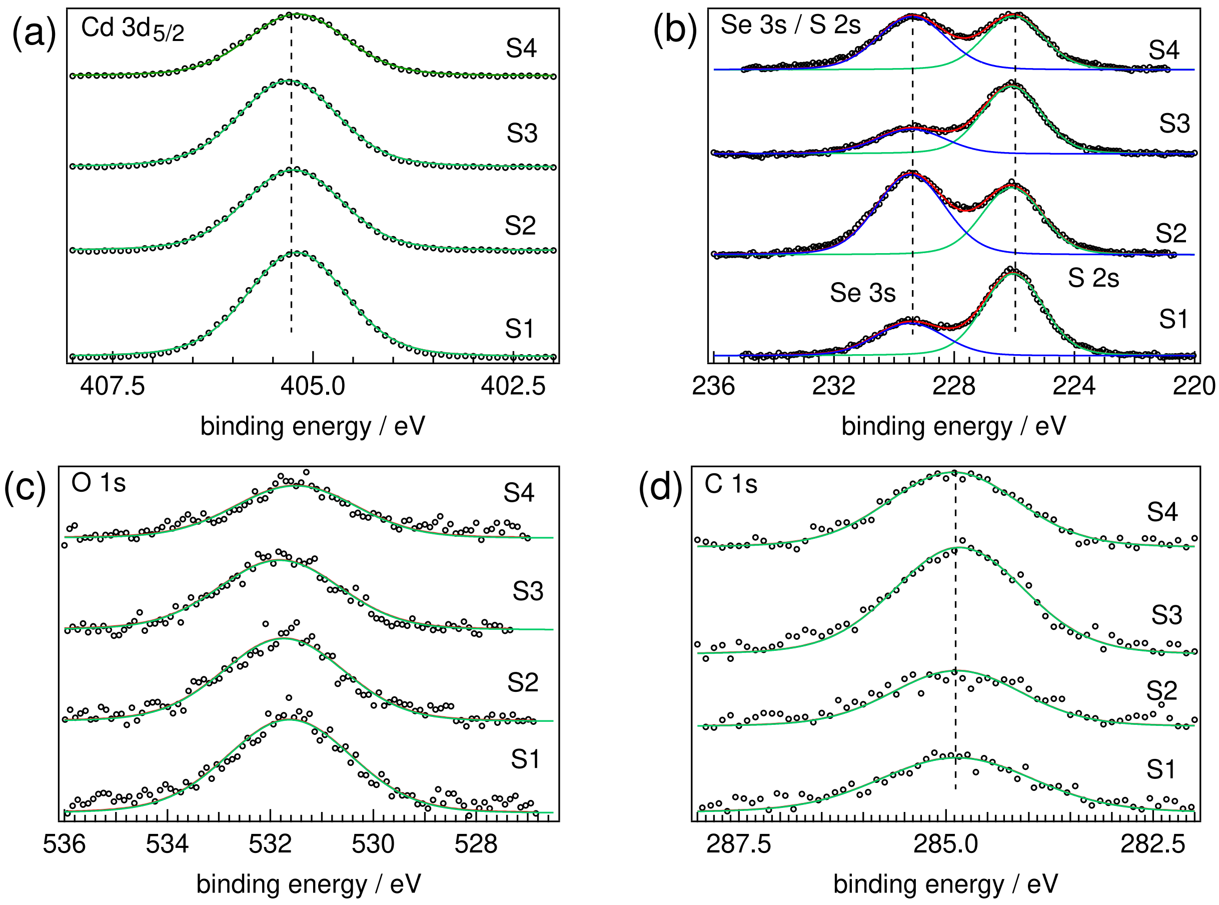


Figure S5: HAXPES data of CdS on PDT ACIGS samples, recorded with an excitation energy of 9 keV: a) Cd $3d_{5/2}$; b) Se 3s/ S 2s; c) O 1s; d) C 1s. The spectra are displayed with the respective fits with Voigt profiles and a linear background subtracted. S1: ACIGS + CsF PDT + 18 nm CdS; S2: ACIGS + CsF PDT + 9 nm CdS; S3: ACIGS + RbF PDT + 18 nm CdS; S4: ACIGS + RbF PDT + 9 nm CdS

Table S1: Relative composition analysis for the C and O contamination for the PDT ACIGS/CdS sample series as determined from HAXPES measurements at both 3 and 9 keV.

| Sample | (C+O)/ACIGS | | (C+O)/CdS | |
|---|---|---|---|---|
| | 3 keV | 9 keV | 3 keV | 9 keV |
| ACIGS + CsF PDT + 18 nm CdS | 1.66e2 | 1.34 | 8.85e-1 | 2.38e-1 |
| ACIGS + CsF PDT + 9 nm CdS | 3.83 | 5.75e-1 | 2.99e-1 | 2.63e-1 |
| ACIGS + RbF PDT + 18 nm CdS | 1.51e2 | 2.14 | 6.47e-1 | 3.7e-1 |
| ACIGS + RbF PDT + 9 nm CdS | 9.85 | 8.99e-1 | 5.91e-1 | 3.54e-1 |

To further investigate how the choice of the metal fluoride PDT influenced the chemical properties at the ACIGS/CdS interface, the binding energy values have been compared for the different samples. A summary of the absorber and buffer related binding energies for the investigated samples are shown in Table S2 and Table 2 (main text) for 3 keV and 9 keV photon energies, respectively. An experimental uncertainty of 0.15 eV shall be considered for all reported binding energy values. The binding energy scale has been aligned to the Cd 4d peak for all samples to exclude charging and surface/interface work function effects (note that In 4d also aligned by this method for all samples). It is likely that the absolute binding energy scale is off by the employed calibration method. However, relative changes between the samples are compared in this work and thus the binding energy values given in Table 2 (main text) and Table S2 shall only be taken as relative.

Table S2: Binding energy positions [eV] recorded with a photon energy of 3 keV for the ACIGS samples investigated. An experimental uncertainty of 0.15 eV shall be considered for all reported binding energy values

| Sample | Cu $2p_{3/2}$ | In $3d_{5/2}$ | Ga $2p_{3/2}$ | Se $3d_{5/2}$ | Ag $3d_{5/2}$ | Cd $3d_{5/2}$ | S $2p_{3/2}$ |
|---|---|---|---|---|---|---|---|
| ACIGS + CsF PDT + 18 nm CdS | - | - | - | - | 367.77 | 405.20 | 161.64 |
| ACIGS + CsF PDT + 9 nm CdS | 932.20 | 444.72 | - | 54.81 | 367.86 | 405.20 | 161.64 |
| ACIGS + RbF PDT + 18 nm CdS | 932.24 | 444.66 | - | - | 367.81 | 405.20 | 161.64 |
| ACIGS + RbF PDT + 9 nm CdS | 932.17 | 444.74 | - | 54.90 | 367.84 | 405.20 | 161.64 |

Figure S6 shows the VB spectra of the investigated samples. The spectra are aligned to Cd 4d as indicated and also discussed in the main text. Similar results are obtained when In 4d is used for binding energy calibration (not shown).

Figure S7 shows the band gap energy for the PDT-AGIGS/CdS samples investigated as determined from Quantum Efficiency measurements.

Figure S8 shows the I-V data for the investigated samples.

Quantum efficiency measurements of the investigated CdS/PDT-ACIGS samples is shown in Figure S9.

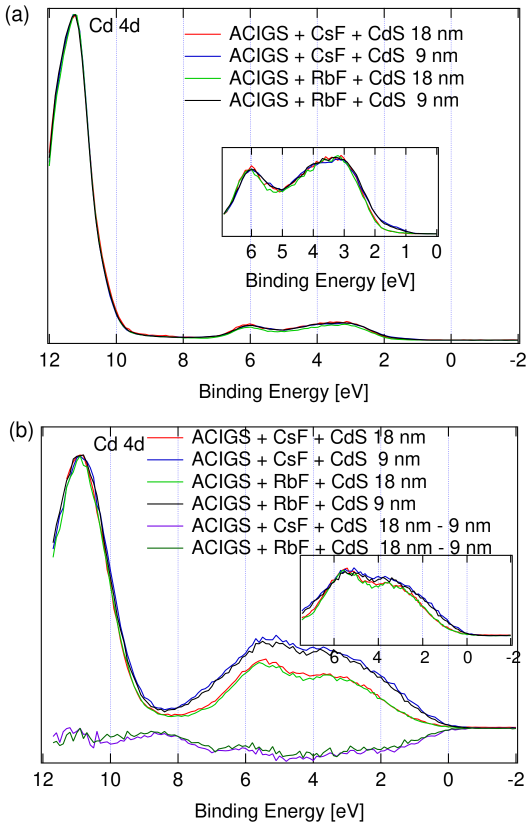


Figure S6: HAXPES VB spectra of the investigated ACIGS/CdS samples recorded with (a) 3 keV, (b) 9 keV photon energy. The spectra have been normalised to the peak intensity of Cd 4d and the binding energy scale has also been aligned to the Cd 4d peak as shown in the figure and mentioned in the main text. Spectra obtained by subtraction of the 9 nm thick CdS from the 18 nm thick CdS at 9 keV are also given for comparison. The insets show a zoom in of the valence band spectra which have been normalised to the maximum peak intensity for comparison.

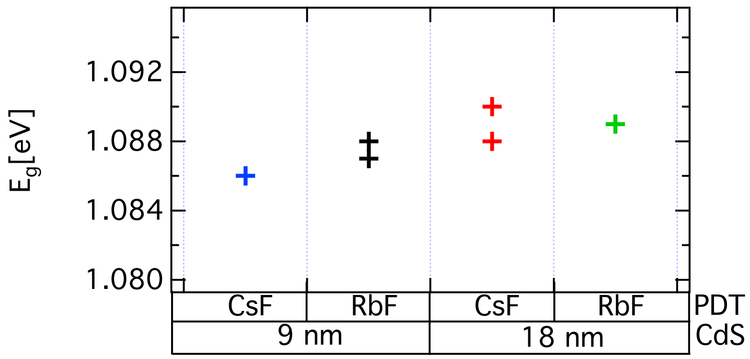


Figure S7: Band gap energy for the investigated samples as determined from Quantum Efficiency measurements.

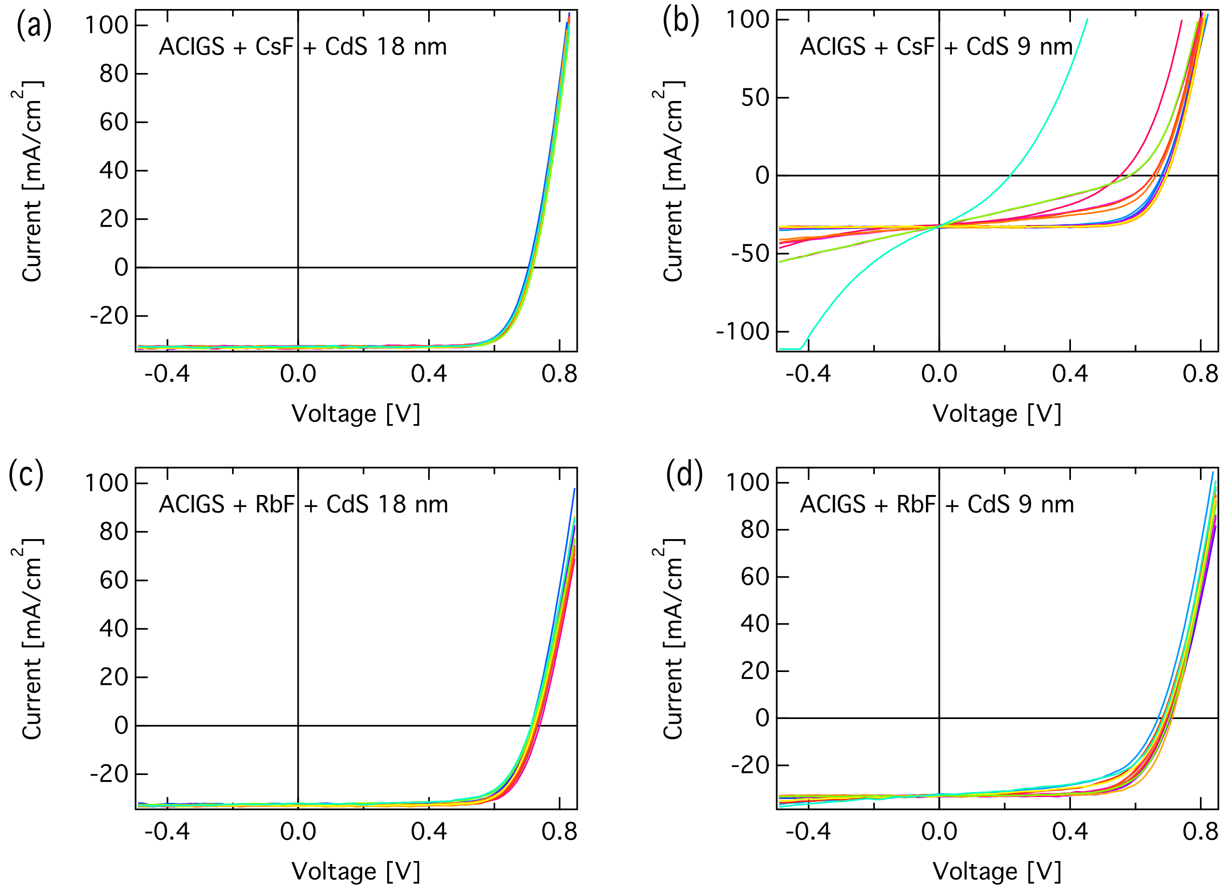


Figure S8: I-V curves for the solar cells fabricated from the PDT-ACIGS/CdS samples investigated in this work (a) ACIGS + CsF PDT + 18 nm CdS; (b) ACIGS + CsF PDT + 9 nm CdS; (c) ACIGS + RbF PDT + 18 nm CdS; (d) ACIGS + RbF PDT + 9 nm CdS. A number of 16 individual cells were measured for each sample.

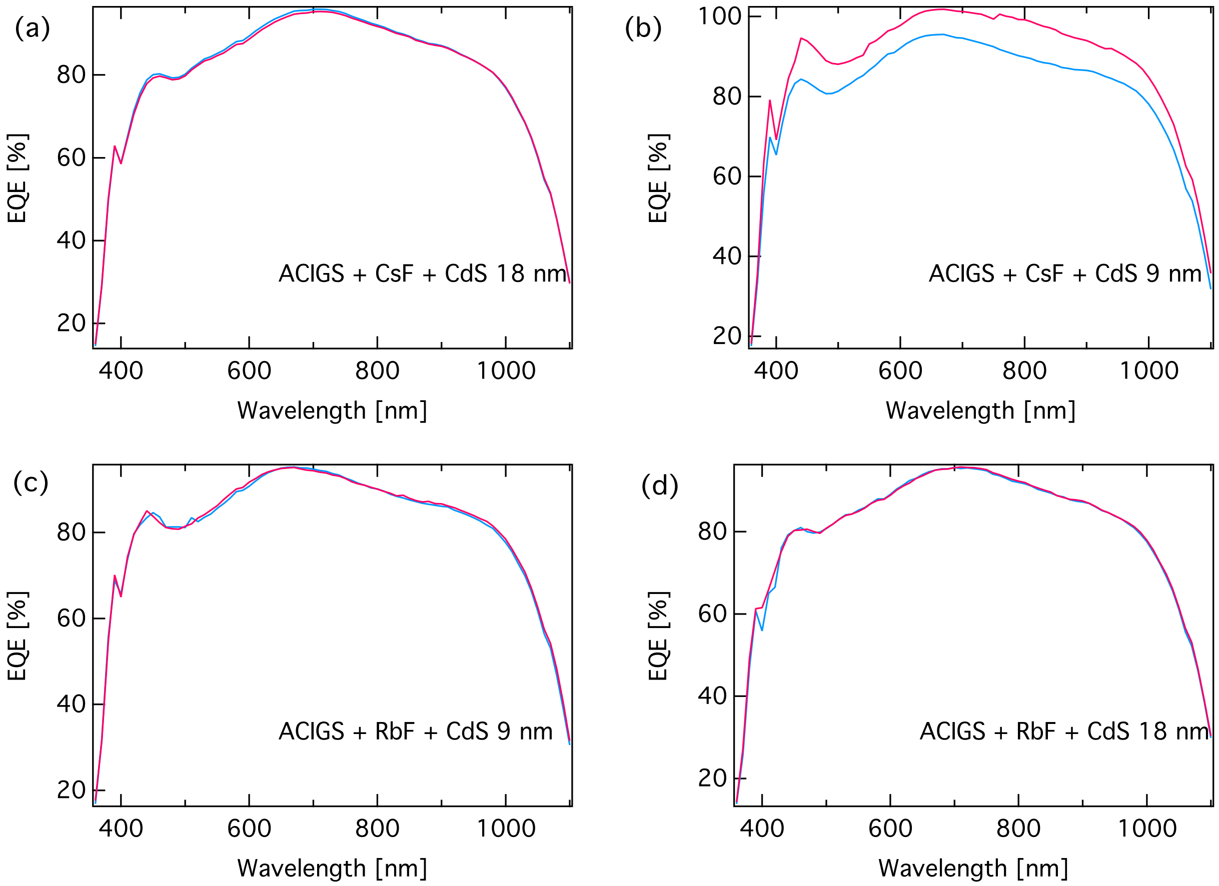


Figure S9: Quantum efficiency measurements for the solar cells fabricated from the PDT-ACIGS/CdS samples investigated in this work (a) ACIGS + CsF PDT + 18 nm CdS; (b) ACIGS + CsF PDT + 9 nm CdS; (c) ACIGS + RbF PDT + 18 nm CdS; (d) ACIGS + RbF PDT + 9 nm CdS.
